# Description of *Meloidoderita salina* sp. n. (Nematoda, Sphaeronematidae) from a micro-tidal salt marsh at Mont-Saint-Michel Bay in France

**DOI:** 10.3897/zookeys.249.4138

**Published:** 2012-12-07

**Authors:** Samad Ashrafi, Didier Mugniéry, Evelyn YJ van Heese, Adriaan C. van Aelst, Johannes Helder, Gerrit Karssen

**Affiliations:** 1 Plant Protection Service, Wageningen Nematode Collection, P.O. Box 9102, 6700 HC Wageningen, The Netherlands; 2 Ghent University, Department of Biology, Ledeganckstraat 35, 9000 Ghent, Belgium; 3 UMR 1349 IGEPP, INRA-Agrocampus Ouest-Université Rennes 1, Bât 320 BP35327 35653 Le Rheu cedex, France; 4 Wageningen University, Department of Plant Science, Laboratory of Nematology, Droevendaalsesteeg 1, 6708 PB Wageningen, The Netherlands

**Keywords:** *Atriplex portulacoides*, cystoid, halophyte, hexagonal, morphology, morphometrics, nematode, new species, sea purslane, SEM, SSU rDNA, taxonomy

## Abstract

*Meloidoderita salina* **sp. n.** is described and illustrated from the halophytic plant *Atriplex portulacoides* L. (sea purslane) growing in a micro-tidal salt marsh in the Mont-Saint-Michel Bay in France. This new species is the first member of *Meloidoderita* Poghossian, 1966 collected from a saline environment, and is characterized by the following features: sedentary mature females having a small swollen body with a clear posterior protuberance; slightly dorsally curved stylet, 19.9 µm long, with posteriorly sloping knobs; neck region irregular in shape and twisted; well developed secretory-excretory (S–E) pore, with markedly sclerotized S-E duct running posteriorly; prominent uterus bordered by a thick hyaline wall and filled with eggs. The adult female transforms into a cystoid. Eggs are deposited in both egg-mass and cystoid. Cystoids of *Meloidoderita salina* **sp.** **n.** display a unique sub-cuticular hexagonal beaded pattern.

Male without stylet, pharyngeal region degenerated, S-E duct prominent, deirids small, developed testis 97.5 µm long, spicules 18.4 µm long, cloacal opening ventrally protruded, small phasmids posterior to cloaca opening and situated at 5.9 (3.2–7.7) µm from tail end, and conical tail ending in a rounded terminus marked with one (rarely two) ventrally positioned mucro. Additionally, some young malesof the new species were observed enveloped in the last J2 cuticle. Second-stage juvenile body 470 µm long, with a 16.4 µm long stylet, prominent rounded knobs set off from the shaft, hemizonid anterior and adjacent to S-E pore, small deirids located just above S-E pore level, genital primordium located at 68–77% of body length, phasmids small and located at about 19 µm from tail tip, and tail 38.7 µm long, tapering to finely pointed terminus with a finger-like projection. Phylogenetic analyses based on the nearly full length small subunit ribosomal DNA sequences of *Meloidoderita salina* **sp. n.** revealed a close relationship of the new species with *Sphaeronema alni* Turkina & Chizhov, 1986 and placed these two species sister to the rest of Criconematina.

## Introduction

Since Poghossian (1966) established the genus *Meloidoderita* Poghossian, 1966 to accommodate the new species *Meloidoderita kirjanovae* Poghossian, 1966, two other *Meloidoderita* species have been described. *Meloidoderita kirjanovae* was isolated from roots of mint (*Mentha longifolia* (L.) Huds.) from the Mergi region in Armenia. Poghossian (1966) placed *Meloidoderita* within Heteroderidae Filipjev & Schuurmans Stekhoven, 1941 (Skarbilovich 1947) on the basis of cyst induction with a pattern of spine-like structures. Wouts and Sher (1971) considered *Meloidoderita* as *genus inquirenda* in the subfamily Heteroderinae Filipjev & Schuurmans Stekhoven, 1941. One year later Wouts (1972) reported that in the previous study, due to a lack of type material and an insufficient description, they “could not establish the exact status of the genus *Meloidoderita*”. Afterwards, after examining five females identified as *Meloidoderita kirjanovae* and on the basis of the presence of a large egg-sac (gelatinous matrix), short stylet, the absence of a cyst, and pronounced galls in the observed roots, Wouts (1972) considered *Meloidoderita* as a valid genus belonging in Meloidogynidae Skarbilovich, 1959 (Wouts 1973).

Kirjanova and Poghossian (1973) re-described *Meloidoderita kirjanovae* and established a newly erected family, Meloidoderitidae, within Criconematidea Taylor, 1936 (1914) (Thorne 1949). Moreover, Poghossian (1975) reported that the material examined by Wouts probably had been contaminated by *Meloidogyne hapla* Chitwood, 1949.

*Meloidoderita kirjanovae* has been recorded parasitizing on *Mentha* spp. (mint and water mint) and *Utrica dioica* L. (common nettle) (Poghossian 1966, Narbaev 1969, Cohn and Mordechi 1982, Vovlas et al. 2006).

Siddiqi (1985, 2000) classified *Meloidoderita* in the subfamily Meloidoderitinae Kirjanovae & Poghossian, 1973, family Sphaeronematidae (Raski & Sher, 1952) Geraert, 1966, superfamily Tylenchuloidea (Skarbilovich, 1974) Raski & Siddiqui, 1975 and suborder Criconematina Siddiqi, 1980.

The second species of *Meloidoderita*, *Meloidoderita safrica*, was described by Van den Berg and Spaull (1982) from soil and root samples of sugarcane (*Saccharum* hybrid) in South Africa.

Golden and Handoo (1984) described *Meloidoderita polygoni* from USA. Previously, Golden (1976) and Andrews et al. (1981) reported the occurrence of a population of *Meloidoderita* sp. from roots of smartweed (*Polygonum hydropiperoides* Michx.), which was not able to infect mint and nettle.

During a nematode survey conducted in Mont-Saint-Michel Bay in France, a *Meloidoderita* population was isolated from soil and roots of the halophyte *Atriplex* (= *Halimione*) *portulacoides* (L.) Aellen. This nematode was infecting roots of sea purslane (*Atriplex portulacoides*) growing in a muddy soil salt marsh region. Preliminary morphological and molecular analyses (G. Karssen, unpublished) indicated that the population differed from all three known described species of *Meloidoderita* and represented a new species. This was the first *Meloidoderita* species collected from a salt marsh environment.

The main objectives of the present study were to:i) describe a new species of *Meloidoderita* isolated from soil and roots samples of *Atriplex portulacoides* from a salt marsh region in France and provide a detailed morphological description based on LM and SEM; ii) characterize *Meloidoderita* species by means of small subunit rDNA sequencing; iii) determine the phylogenetic position of *Meloidoderita* within the suborder Criconematina.

## Materials and methods

### Collection of samples

Soil and root samples were isolated from *Atriplex portulacoides* grown in muddy soil of a costal tidal salt marsh environment in “Le Vivier- sur- Mer” at 48°36'32"N and 1°47'00"W at Mont-Saint-Michel Bay in France.

The Mont-Saint-Michel Bay (MSMB) is a costal embayment and macro-tidal environment located on the English Channel (Southern gulf of Normandy) between the Cotentin Peninsula and the Brittany coast, in the northwestern coast of France (Detriche et al. 2011, Dubois et al. 2007). The climate is Oceanic-Breton with average annual temperature of 9˚C (Costil et al. 2001). Samples were collected during the months of March, June, September, and December in 2007. The average salinity of soil in MSMB is about 34–35 g/L (3.5%). The tides cover the area where *Atriplex portulacoides* grows about twenty times a year.

The Mont-Saint-Michel Bay is a specific ecosystem on a small geographic scale. Despite the presence of numerous ecological studies that have been applied since 1979 in MSMB, nematodes have been mostly neglected (Lefeuvre et al. 2003).

### Nematode extraction and comparison

To obtain a homogenized sample of the cohesive muddy soil, we gently mixed samples in a kneading machine for 15 min. Afterwards, nematodes including juveniles, males, cystoids, and eggs, were extracted from soil samples by means of a magnesium sulphate centrifugal flotation technique (Coolen 1979).

Females were collected with two different methods: i) centrifugal flotation method (Coolen 1979) for extracting females, and ii) direct handpicking of females and egg-masses from roots with the aid of dissecting tools under a stereomicroscope. Root samples were washed with tap water under low pressure to prevent damage to the nematodes.

The *Meloidoderita* populations and a *Sphaeronema* Raski & Sher, 1952 population used for comparison are listed in Table 1.

**Table 1.** Host and origin of the populations of three *Meloidoderita* species and one *Sphaeronema* species which were compared with the population of *Meloidoderita salina* sp. n.

Species	Host	Origin
*Meloidoderita kirjanovae* (Poghossian, 1966) Kirjanova & Poghossian (1973)	Mentha longifolia (L.) Huds.	Megri region, Armenia
*Meloidoderita kirjanovae* characterized by Golden and Handoo (1984)	Mentha longifolia	Mediterranean region
*Meloidoderita kirjanovae* characterized by Siddiqi (1985)	Mentha longifolia	Armenia
*Meloidoderita kirjanovae* characterized by Vovlas et al. (2006)	Mentha aquatic L.	Laceno Lake at Avellino, southern Italy
*Meloidoderita safrica* Van den Berg & Spaull, 1982	Saccharum hybrid (Sugar cane)	Mposa area of Natal, South Africa
*Meloidoderita polygoni* Golden & Handoo, 1984	Polygonum hydropiperoides Michx.	Beltsville, Maryland, USA
*Sphaeronema alni* Turkina & Chizhov, 1986 (topotype population)	Alnus incana (L.) Moench, A. glutinosa L., Betula pubescens Ehrh.	Russia

## Light and scanning electron microscopy

Specimens for light microscopy (LM) were fixed in heated (70^°^C) TAF (2 ml triethanolamine, 7ml formaldehyde and 91 ml distilled water (Courtney et al. 1955)), and processed to anhydrous glycerin following the method of Seinhorst (1966). Fixed specimens including second-stage juveniles, males, females, cystoids, egg-masses and eggs were mounted in a small drop of desiccated glycerin with the paraffin wax method on Cobb slides (Southey 1986).

Measurements and drawings were performed on a light microscope Olympus BH-2 equipped with Nomarski Differential Interference Contrast (DIC).

Specimens were drawn with a drawing tube, scanned and modified using Photoshop software version CS 5.1.

Light micrographs of specimens were taken with a Leica DC 300 F camera attached to a Zeiss Axio Imager M1 microscope. The original descriptions of closely related species (Table 1) were used for morphological and morphometrical comparison.

For SEM observation nematodes were fixed in 3% glutaraldehyde buffered with 0.05M phosphate buffer (pH 6.8) for 1.5 h and post-fixed with 2% osmium tetroxide for 2h at 22^°^C. The specimens were dehydrated in a seven-graded ethanol series of 15-25-35-50-70-95 and 100% (Wergin 1981), critical point dried with carbon dioxide, and sputter coated with a layer of 4–5 nm Pt in a dedicated preparation chamber (CT 1500 HT, Oxford Instruments). The nematodes were examined and photographed with a field emission electron microscope Jeol 6300 F, at 5 kV (Karssen 1996, 1998).

## DNA Extraction, PCR-Based amplification, Cloning and Sequencing

Single nematodes (five individuals in total) were transferred to a 0.2 ml Eppendorf vial containing 25 µl of sterile water. An equal volume of lysis buffer containing 0.2 M NaCl, 0.2 M Tris-HCl (pH 8.0), 1% (vol/vol) β-mercaptoethanol, and 800 µg/ml of proteinase K was added. Lysis took place in a Thermomixer (Eppendorf, Hamburg, Germany) at 65°C and 750 rpm for 2 h followed by a 5 min incubation at 100°C (to inactive proteinase). Lysate was immediately used or stored at –20°C. SSU rDNA was amplified as two partially overlapping fragments using three universal and one nematode-specific primer (1912R). The latter was included to avoid amplification of non-target eukaryotic SSU rDNA. For the first fragments, either the primer 988F (5'-ctc aaa gat taa gcc atg c-3') or the primer 1096F (5'-ggt aat tct gga gct aat ac-3') was used in combination with the primer 1912R (5'-ttt acg gtc aga act agg g-3'). The second fragment was amplified with primers 1813F (5'-ctg cgt gag agg tga aat-3') and 2646R (5' -gct acc ttg tta cga ctt tt-3'). PCR was performed in a final volume of 25 µl containing 3 µl of 100 times-diluted crude DNA extract, 0.1 µM of each PCR primer and a ready-To-Go PCR bead (GE Healthcare, Little Chalfont, UK). The following PCR program was used: 94°C for 5 min; 5× (94°C, 30 s; 45°C, 30 s; 72°C, 70 s) followed by 35× (94°C, 30 s; 54°C, 30 s; 72°C, 70 s), and 72°C for 5 min. Gel-purified amplification products (Marligen, Ijamsville, MD) were cloned into a TOPO-TA vector (Invitrogen, Carlsbad, CA) and sent off for sequencing using standard procedures (Holterman et al. 2009). The newly generated SSU rDNA sequences were deposited at GenBank under accession numbers FJ969126 and FJ969127.

## Sequence alignment

SSU rDNA-obtained sequences were aligned using the ClustalW algorithm as implemented in the program BioEdit 7.0.1 (Hall 1999). Manual improving and editing the alignment was then performed using arthropod secondary structure information (http://www.psb.ugent.be/rRNA/secmodel/index.html) according to Wuyts et al. (2000). Outgroup taxa and those nematodes compared with the sequence of the new *Meloidoderita* were chosen in accordance with Holterman et al. (2009). The final alignment included 39 SSU rDNA sequence and contained 1883 aligned position including gaps.

## Phylogenetic analyses

The phylogenetic tree was constructed using Bayesian inference (MrBayes 3.1.2 (Ronquist and Huelsenbech 2003)) and a fast maximum likelihood method (RAxML-VI- HPC v.4.0.0 (Stamatakis 2006)). Modeltest 3.06 (Posada and Crandall 1998) identified the general time reversible (GTR) model with invariable sites and a gamma-shaped distribution of substitution rates as the best substitution model. Bayesian analysis was performed with a random starting tree and four Markov chains. The programme was run for 5 × 10^6^ generations with a sampling frequency of 1,000 generations. Two independent runs were performed for each analysis. After discarding the ‘burn-in’ samples of 500,000 generations, sampled trees were combined to generate a 50% majority rule consensus tree, which represents posterior probabilities.

The second phylogenetic tree was constructed with a fast maximum likelihood method. The SSU rDNA alignment was analysed at a distant server (CIPRES, http://www.phylo.org) running the program, RAxML-VI-HPC v.4.0.0 using the same GTR model. One hundred bootstrap replicates were performed.

## Results

### Meloidoderita salina sp. n.

urn:lsid:zoobank.org:act:02A22EB6-85D4-4783-98AB-A6FA894EEAAD

http://species-id.net/wiki/Meloidoderita_salina

**Figure 1.**
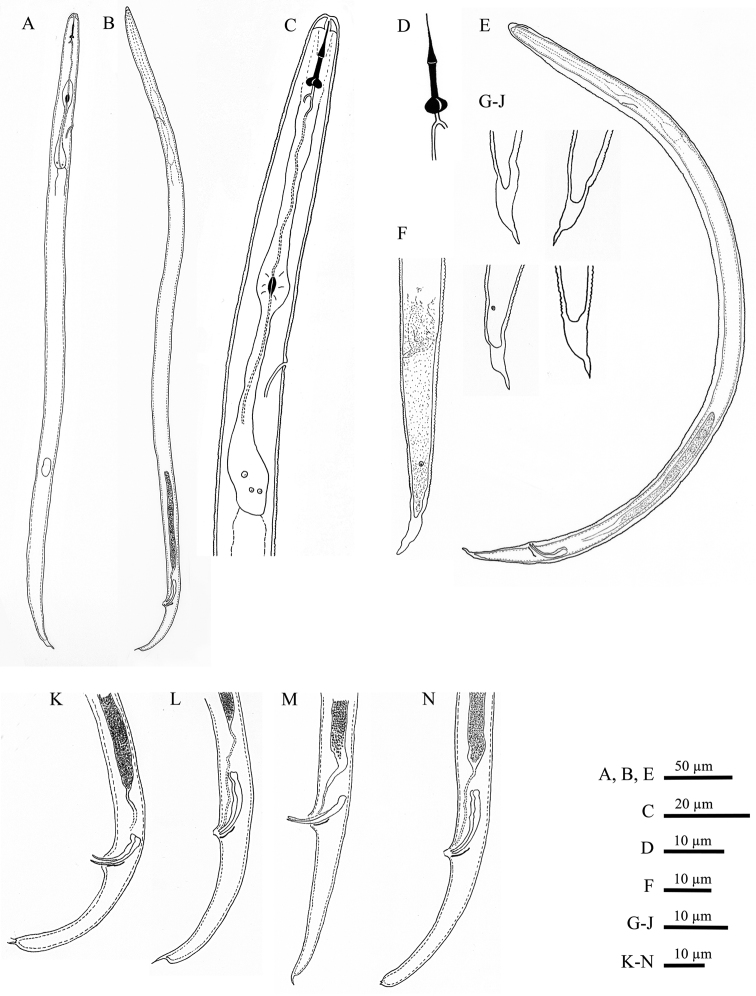
*Meloidoderita salina* sp. n. **A** Second-stage juvenile (J2) **B** Male **C** J2 anterior region **D** J2 stylet **E** Male within old J2 cuticle **F** J2 posterior region **G–J** J2 Tail tip **K–N** Male posterior region.

Figs 1 [Fig F2] [Fig F3] [Fig F4] [Fig F5] [Fig F6] [Fig F7] –8 Table 2

#### Measurements.

*Females, males and second-stage juveniles*: See Table 2. *Embryonated eggs (n= 44)*: Length: 102.5 ± 5.0 (94.4–112) µm; diam.: 41.7 ± 1.9 (38.4–46.4) µm; length/width ratio: 2.5 ± 0.2 (2.1–2.9). *Cystoids (n=18)*: Length: 224 ± 34.5 (176–336) µm; Width: 187.5 ± 33.1 (145.6–280) µm; length/width ratio: 1.2 ± 0.1 (1.0–1.7).

#### Description.

**Figure 2. F2:**
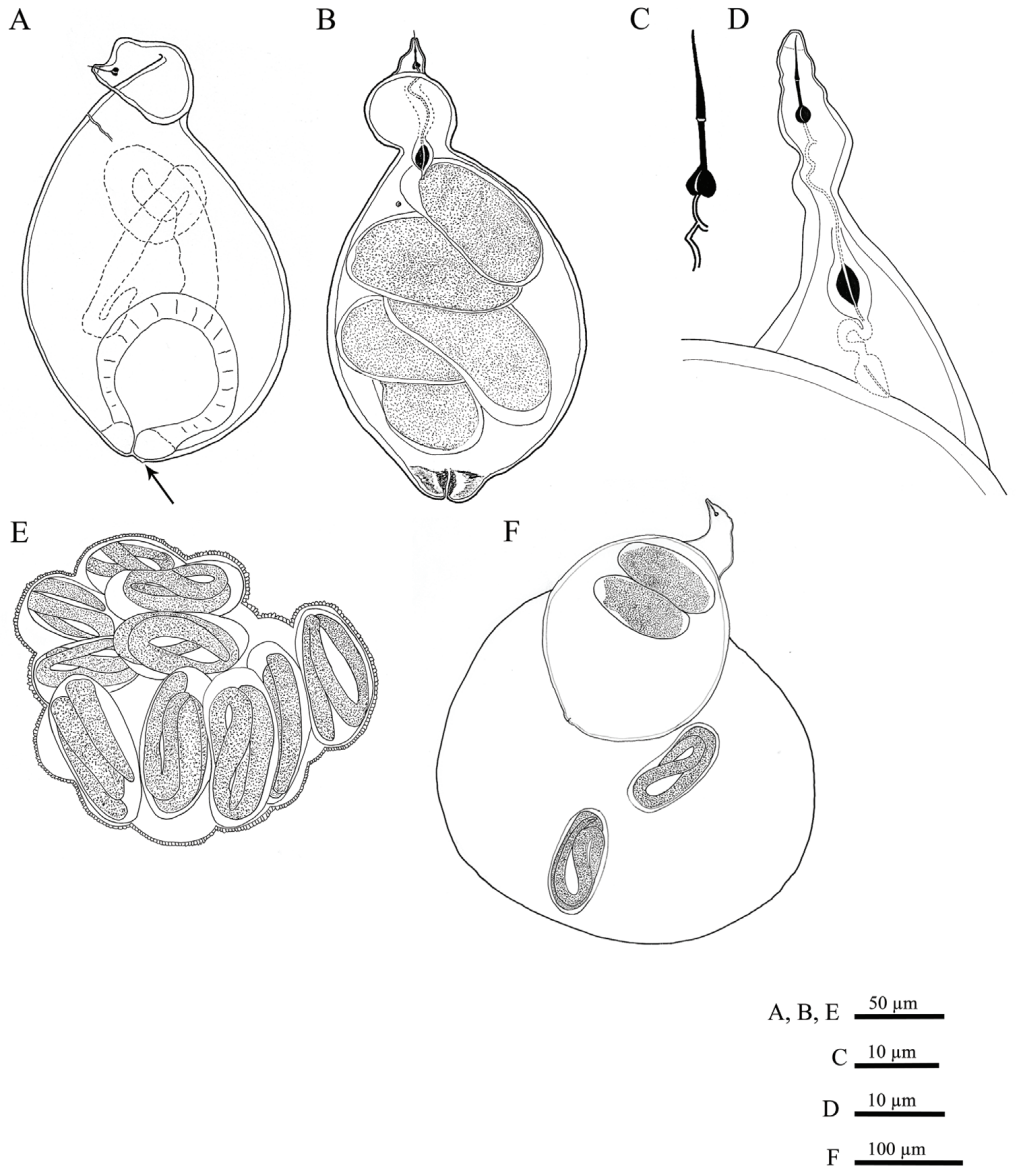
*Meloidoderita salina* sp. n. **A, B** Female body (arrow = anus) **C** Female stylet **D** Female neck region **E** Cystoid **F** Female with egg-mass.

**Female.** Body swollen with a small posterior protuberance, pearly white to light brown, oval to pear-shaped. Neck region distinct, irregular shaped, usually twisted, 49 to 82 µm in length (Figs 2, 8). Body cuticle thick, without annulation. Head continuous with body, without annules. Cephalic framework weakly developed, lip region flattened. Stylet well developed, with posteriorly sloping oval-shaped knobs; stylet cone longer than shaft, slightly curved dorsally, shaft cylindrical (Fig. 2C). Dorsal gland orifice (DGO) close to basal knobs; vestibule extension visible. Secretory-excretory (S-E) pore well developed with clear cuticular lobes, located posterior to the neck, about 35 (20–56)% from anterior end of body; S-E duct markedly sclerotized, running posteriorly. Pharyngeal lumen from stylet to valve of metacorpus prominent. Metacorpus usually oval-shaped, situated at the posterior part of neck region, with distinct sclerotized valve apparatus, distance from middle of metacorpus to anterior end about 58 ± 10 µm long. Posterior gland bulb extending into anterior portion of swollen body cavity. Reproductive system extending towards pharyngeal region, monodelphic, spermatheca not observed; vulva with noticeable protruding lips, positioned usually at the posterior extremity of the body, rarely subterminal. Vulval lips forming thickened and muscular area around vulval slit (vulval area). Anus faint, opening pore-like, difficult to observe by LM, located at the base of dorsal vulval lip, apparently not functional (Figs 5E, 8C). Uterus swollen, prominent, bordered by a thick hyaline wall, becoming enlarged and filled with eggs, transforming into a cystoid within the female cuticle.

**Male.**Body slender, vermiform, tapering at both ends but more posteriorly, usually slightly curved ventrally at tail region. Cuticle marked by fine annulations, about 0.9 µm wide. Young males usually still enveloped in the last cuticle of second-stage juveniles (Fig. 4D). Lateral field beginning with 2 weak lines, roughly between head end and S-E pore level, and continuing with four weak lines behind S-E pore level. Head continuous with body, rounded-conoid, without annules and separated lips, distinct but weak cephalic framework present; amphidial apertures slit-like, angled, adjacent to oral opening surrounded by a small elevated oral disc (Fig. 7B). Pharyngeal region degenerated except for the posterior bulb, no stylet observed. S-E pore well developed, adjacent to hemizonid. S-E duct strongly sclerotized anteriorly (Fig. 4E). Deirids small, located just above S-E pore level (Fig. 7C). Monorchic, outstretched, testis well developed, with small vas deferens about6 µm long. Spicules paired, equal, not fused, arcuate, with rounded manubrium. Gubernaculum slightly curved. Cloacal tube about 2 µm long. Bursa-like structure visible by SEM (Fig. 7E). Phasmids small, posterior to cloacal opening. Tail conical, tapering to rounded terminus, marked with one or rarely two mucrones; if two are present, ventral mucro usually smaller; terminal mucro positioned ventrally, length 0.6‒3.2 µm (Fig. 1K–N).

**Figure 3. F3:**
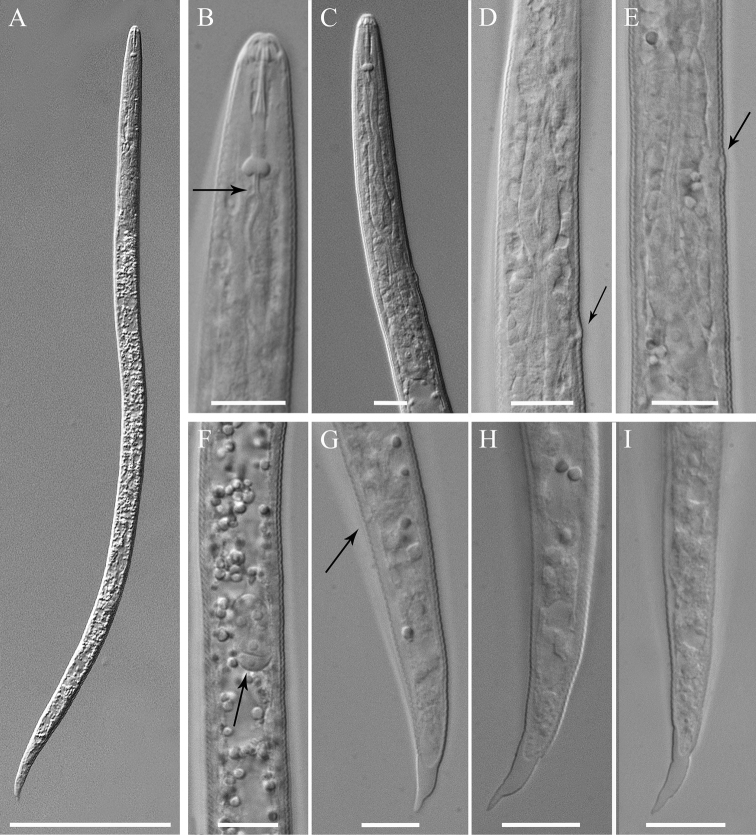
*Meloidoderita salina* sp. n. LM photographs of second-stage juveniles. **A** Entire body **B, C** Anterior body (arrow =DGO) **D** S-E duct adjacent to hemizonid (arrow = S-E duct) **E** Basal bulb (arrow = hemizonid) **F** Mid-body portion (arrow = primordium) **G-I** Tail (arrow = anus). Scale bars: **A** =100 µm **B–I** = 10 µm.

**Second-stage juvenile.** Body slender, vermiform, tapering at both ends but more so posteriorly, slightly ventrally curved at tail region; cuticle with fine annulations, annules about 1 µm wide. Lateral field with two visible outer lines in some specimens; in SEM, lateral field starts with three lines about 30 µm from head at neck region, four lines at 20%, and five lines at 33% of body length. Head continuous with body, rounded-conoid with slightly elevated concave oral disc, with distinct but relatively weak cephalic framework, without annules; two open slit-like amphidial apertures adjacent to slightly elevated concave oral disc surrounding the oral aperture, as visible by SEM (Fig. 6A). Lips not visible as distinct structures. Stylet well developed; cone tapering towards fine point; shaft straight; knobs rounded, prominent, sloping slightly posteriorly, set off from shaft (Fig. 1D). DGO close to stylet base. Metacorpus slightly elongated, with weak valves. S-E pore posterior and adjacent to hemizonid, located at isthmus level; hemizonid 2–3 annules long (Fig. 3D). Isthmus slender, distinct. Pharyngeal glands slightly overlapping intestine ventrolaterally. Deirids small, located just above S-E pore level. Genital primordium located posteriorly at 68‒77% of body length. Anus small, weakly developed, obscure by LM, pore-like (Fig. 6E). Phasmids small, difficult to observe by LM, located at about 19 µm from tail tip. Tail conical, slightly curved ventrally, tapering to finely pointed terminus, with finger-like projection. Hyaline tail part clearly delimitated anteriorly (Fig. 3G–I).

**Cystoid.** Irregularly spherical to oval, filled with embryonated and non-embryonated eggs. Colour ranging from light in young cystoids to brown in older cystoid bodies. Body wall thickness 5.3 ± 1.2 (3.2–8.3) µm, containing bead-like outgrowths, displaying a specific sub-cuticular hexagonal beaded pattern (Figs 5, 8).

**Egg mass.**Females and cystoids usually completely surrounded by a gelatinous matrix (egg-mass) measuring about 316 ± 71.0 µm in length and 275 ± 54.0 µm in diameter (Fig. 5F).

**Eggs.** Oblong, translucent, egg shell without any visible markings, enveloped in a gelatinous matrix or within a cystoid.

#### Type host and locality.

Collected from rhizosphere and roots of the salt marsh halophytic shrub *Atriplex portulacoides* L. (= *Halimione portulacoides* (L.) Aell.), the most abundant species in ungrazed European salt marshes (Bouchard et al. 1998), growing in cohesive muddy soil of the macro-tidal salt marshes of ‘Le Vivier-sur-Mer’ at 48°36'32"N latitude and 1°47'00"W longitude at Mont-Saint-Michel bay, France.

**Table 2.** Morphometrics of *Meloidoderita salina* sp. n. All measurements are in µm and in the form: mean ± SD (range).

Character	Female		Male Paratypes	J2 Paratypes
	Holotype	Paratypes		
n	-	43	21	27
L	286	260 ± 34 (186–358)	469 ± 28 (416–522)	471 ± 19 (419–496)
a	1.8	1.3 ± 0.2 (0.9–1.8)	40.0 ± 2.8 (35.0–45.0)	30.4 ± 1.1 (28.2–32.5)
b	-	-	4.1 ± 0.4 (3.3–4.8)	3.7 ± 0.2 (3.4–4.3)
c	-	-	12.9 ± 1.4 (11.1–15.9)	12.2 ± 0.9 (9.9–13.9)
c´	-	-	3.9 ± 0.5 (2.5–4.6)	4.2 ± 0.2 (4.0–4.3)
Greatest body diam.	152	206 ± 37 (126–320)	11.8 ± 0.8 (10.9–13.4)	15.5 ± 0.5 (14.1–16.0)
Body diam. At excretory pore	-	-	10.4 ± 1.1 (7.7–12.8)	14.4 ± 0.5 (13.4–15.4)
Body diam. at anus or cloacal opening	-	-	9.6 ± 0.9 (7.0–10.9)	9.2 ± 0.6 (8.3–10.9)
Head region height	-	-	2.2 ± 0.3 (1.9–2.6)	4.0 ± 0.2 (3.8–4.5)
Head region diam.	-	-	3.7 ± 0.4 (3.2–4.5)	7.0 ± 0.4 (6.4–7.7)
Stylet length	19.2	19.9 ± 0.7 (19.0–22.0)	-	16.4 ± 0.5 (14.7–17.3)
Stylet cone	12	11.6 ± 0.6 (10.5–12.8)	-	-
Stylet shaft	-	-	-	5.1 ± 0.3 (4.5–5.8)
Stylet knob height	2.6	3.0 ± 0.4 (2.6–4.0)	-	2.6 ± 0.2 (1.9–3.2)
Stylet knob width	3.2	3.7 ± 0.5 (3.2–5.0)	-	3.7 ± 0.2 (3.2–3.8)
Ant. end to knobs base	-	-	-	18.4 ± 0.4 (17.3–19.2)
DGO	3.2	3.3 ± 0.5 (2.5–4.0)	-	2.4 ± 0.4 (1.9–3.2)
Ant. end to metacorpus	42.9	-	-	65 ± 1.2 (63–67)
Metacorpus valve length	16.0	15.8 ± 0.9 (15.0–17.9)	-	-
Metacorpus valve width	8.9	8.5 ± 0.8 (7.7–10.0)	-	-
Pharynx length	-	-	115 ± 13 (90–138)	126 ± 7 (111–144)
Ant. end to excretory pore	74	92 ± 22.1 (55–125)	82 ± 5.5 (74–96)	87 ± 3.0 (77–93)
Ant. end to genital primordium	-	-	-	340 ± 20 (305–371)
Genital promordium to posterior end	-	-	-	131 ± 12 (105–150)
Genital primordium length	-	-	-	13.0 ± 1.3 (9.6–15.4)
Genital primordium width	-	-	-	6.8 ± 1.0 (4.5–9.0)
Tail length	-	-	36.6 ± 3.8 (27.5–41.6)	38.7 ± 2.5 (33.9–44.2)
Hyaline tail terminus	-	-	-	8.1 ±1.0 (6.4–9.6)
Phasmid to posterior end	-	-	5.9 ± 1.5 (3.2–7.7)	-
Spicule length	-	-	18.4 ± 1.8 (15.4–21.1)	-
Gubernaculum length	-	-	5.3 ± 0.5 (4.5- 6.4)	-
Testis	-	-	98 ± 21.9 (62- 137)	-
Vulva slit length	20.4	19.5 ± 1.4 (16.0–22.5)	-	-
Vulva-anus	16.0	17.3 ± 2.6 (13.4–23.0)	-	-
Vulva area length	-	41.0 ± 4.9 (32.0–54)	-	-
Vulva area diam.	-	32.4 ± 3.7 (25.6–40.0)	-	-
Cuticle thickness	3.2	5.0 ± 1.4 (2.5–7.7)	-	-
(Excretory pore/L)*100	-	-	17.5 ± 0.8 (16.2–18.9)	18.6 ± 0.8 (17.1–20.6)
Genital primordium % of body length	-	-	-	72.1 ± 2.6 (68.2–77.2)
Hyaline % of tail length	-	-	-	21.0 ± 3.0 (15.1–26.3)

**Figure 4. F4:**
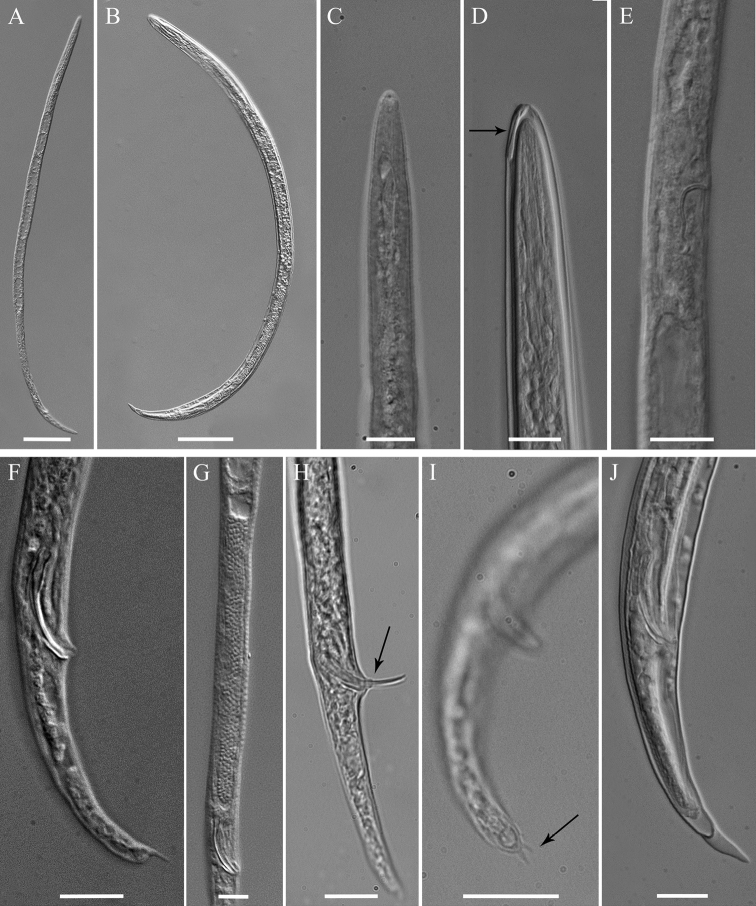
*Meloidoderita salina* sp. n. LM photographs of males. **A** Entire body **B** Male within the second-stage juvenile (J2) cuticle **C** Anterior body **D** Anterior body of male within the old cuticle of J2 (arrow = anterior portion of J2 stylet) **E** S-E duct **F** Posterior region **G** Testis **H** Spicule and cloacal tube (arrow) **I** Tail tip (arrow = mucron) **J** Posterior end of male within the old cuticle of J2. Scale bars: **A, B** = 50 µm **C–J** = 10 µm.

**Figure 5. F5:**
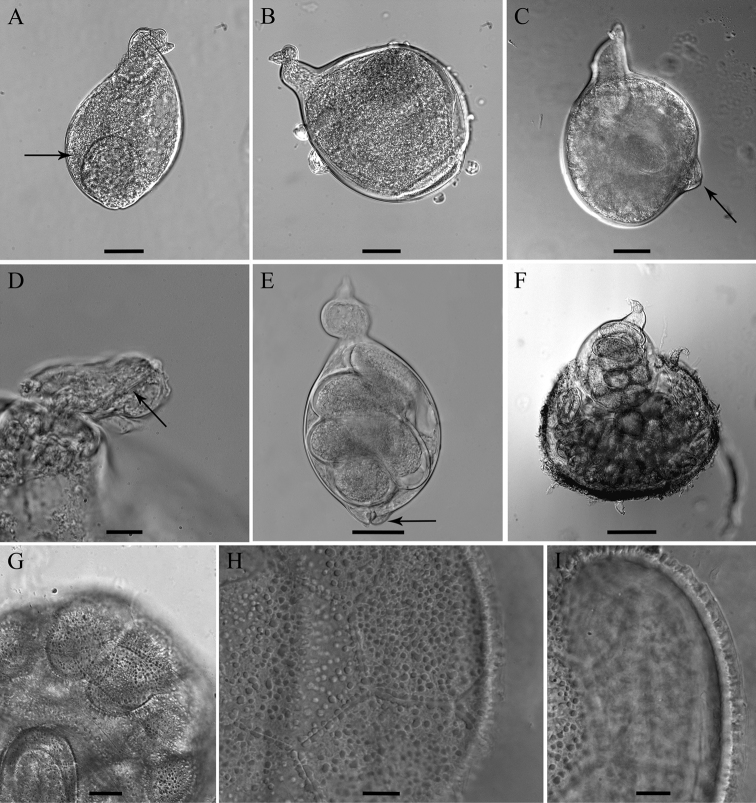
*Meloidoderita salina* sp. n. LM photographs of females. **A, B** Entire body (arrow = uterus) **C **Sub-terminal protruded vulva (arrow) **D** Head region (arrow = stylet) **E** Entire body (arrow = vulva) **F** Female surrounded by egg-mass **G** Cystoid **H, I** Hexagonal beaded pattern. Scale bars: **F**= 100 µm **A–C, E** = 50 µm **D, G–I** = 10 µm.

#### Type material.

Holotype female (slide WT 3591) and paratypes (second-stage juveniles, females, cystoids and males) (slides WT 3592-WT 3595) deposited in the Wageningen Nematode Collection (WaNeCo), Wageningen, The Netherlands. Additional second-stage juvenile, female, cystoid and male paratypes deposited at each of the following collections: Biology Department, Gent University, Gent, Belgium; Central Science Laboratory (CSL), Sand Hutton, York, UK.

#### Etymology.

The specific epithet refers to salty soil (saline environment) and is derived from the Latin word sal or salis meaning “salt”.

#### Diagnosis and relationships.

*Meloidoderita salina* sp. n. is characterized by sedentary mature females having a small swollen body with a clear posterior protuberance, stylet 19.9 (19–22) µm long, stylet cone slightly curved dorsally and longer than shaft, with posteriorly sloping knobs, neck region irregular in shape and twisted, well developed S-E pore, prominent uterus bordered by a thick hyaline wall and filled with eggs. *Meloidoderita salina* sp. n. is further distinguished by the cystoid having a unique sub-cuticular hexagonal beaded pattern.

Male without stylet, pharyngeal region degenerated, S-E duct prominent, spicules 18.4 (15.3–21.1) µm long, deirids just above S-E pore level, small phasmids posteriorly to cloaca opening and situated at 5.9 (3.2–7.7) µm from tail end, conical tail ending in a rounded terminus with one (rarely two) ventrally positioned mucro.

Second-stage juvenile body is 470 (419–496) µm long, with a 16.4 (14.7–17.3) µm long developed stylet, prominent rounded knobs set off from the shaft, hemizonid anterior and adjacent to S-E pore, tail 38.7 (33.9–44.2) µm long tapering to a finely pointed terminus with a finger-like projection.

On the basis of morphology, the female of *Meloidoderita salina* sp. n. resembles other species of the genus (*Meloidoderita kirjanovae*, *Meloidoderita safrica* and *Meloidoderita polygoni*) in the shape of the neck region (twisted, irregular and variable in size), the shape of the vulva (protruded), and the shape of the uterus (prominent, with large cells and a thick wall). Males of the four species are similar in lack of a stylet, degenerated pharyngeal region, the shape of the spicules (arcuate), the shape of the cloacal opening (ventrally protruded), and the shape of the tail (slightly curved ventrally, ending in a terminal mucro). Second-stage juveniles have a continuous head region, weakly sclerotized cephalic framework, similar shape of the tail (conically tapering to a pointed terminus, often with a finger-like terminal mucro), obscure anus, and position of hemizonid (anterior and adjacent to S-E pore).

*Meloidoderita salina* sp. n. differs from the previously described species by a smaller female body, a longer J2 body, the male with a longer body length and (except *Meloidoderita kirjanovae* described by Poghossian (1975)) by the present of a bursa-like structure, and by having a smaller cystoid body with a unique body cuticle surface pattern (displaying a hexagonal beaded pattern *vs* a spine-like structure in *Meloidoderita kirjanovae*, *Meloidoderita polygoni* and *Meloidoderita safrica*). It also differs from them in known hosts and the saline habitat.

The new speciesdiffers in other characters from *Meloidoderita kirjanovae* by females having a longer stylet length and a much shorter distance from anus to vulval slit. Male differs from those characterized by Golden and Handoo (1984), and Vovlas et al. (2006) by having longer spicules length (15.4–21.1 *vs* 13.4–16.1, and 13–15 µm, respectively), and by a lateral field with 2–4 *vs* 3 incisures, and 4 incisures in *Meloidoderita kirjanovae* as redescribed by Kirjanova and Poghossian (1973). The second-stage juvenile of *Meloidoderita salina* sp. n. differs from *Meloidoderita kirjanovae* characterized by Golden and Handoo (1984), Siddiqi (1985) and Vovlas et al. (2006) in having a longer stylet (14.7–17.3 *vs* 12.9–14, 12–14, and 12–15 µm, respectively), lateral field (with 3–5 *vs* 3 incisures), a shorter hyaline tail with 6.4–9.6 µm long *vs* 8.1–13.3 µm long in those reported by Golden and Handoo (1984), 9–14 µm long in Siddiqi (1985), and 14–15 µm long in those of *Meloidoderita kirjanovae* re-described by Kirjanova and Poghossian (1973). Second-stage juveniles also differ from those reported by Golden and Handoo (1984) and Vovlas et al. (2006) by a shorter tail (33.9–44.1 *vs* 38–51, and 41–50 µm, respectively).

*Meloidoderita salina* sp. n. differs from *Meloidoderita safrica* by the female having DGO closer to base of stylet (2.5–4.0 *vs* 8.1–22.1µm), shorter distance from vulval slit to anus (13.4–23.0 *vs* 22.4–24.3 µm), by the male having a shorter testis (62–137 *vs* 190–319 µm), and by the J2 having a longer distance from anterior end to base of pharynx (111–144 *vs* 51.8–75.4 µm).

It differs from *Meloidoderita polygoni* females having a longer stylet (19.0–22.0 *vs* 15.0–17.4 µm), shorter distance from vulval slit to the anus (13.4–23.0 *vs* 32.0–86 µm), and a shorter vulval slit (16.0–22.5 *vs* 22.0–34.0 µm), and by the male without stylet *vs* visible anterior stylet part, a shorter tail (27.5–41.6 *vs* 32.0–56).

The new species is morphologically close related to the genus *Sphaeronema*, particularly to *Sphaeronema alni* Turkina & Chizhov, 1986. According to their observed phylogenetic relationships, they form together a highly supported clade. The absence of a cystoid stage in *Sphaeronema* is the most import differences compared to *Meloidoderita*.Additionally *Meloidoderita salina* sp. n. differs from *Sphaeronema alni* by females having a head region continuous with body *vs* head cap set off from neck and the lip region lacking annulations *vs* 2 annuli. The second-stage juveniles has a tail conically tapering to a pointed terminus, often with a finger-like projection, whereas in *Sphaeronema alni* the tail tapers gradually to a finely rounded terminus.

**Figure 6. F6:**
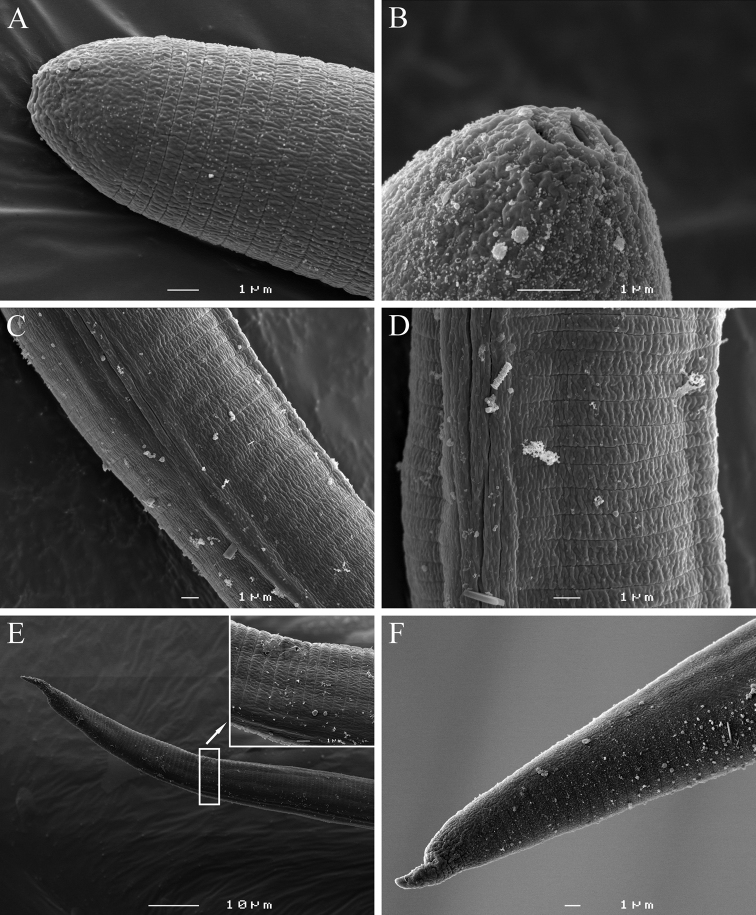
*Meloidoderita salina* sp. n. SEM photographs of second-stage juveniles. **A** Lateral view of head region **B** Amphids **C** Lateral field at 30 µm from anterior end **D** Lateral field at 33% of body length **E** Posterior region (arrow = anus) **F** Lateral view of tail region.

**Figure 7. F7:**
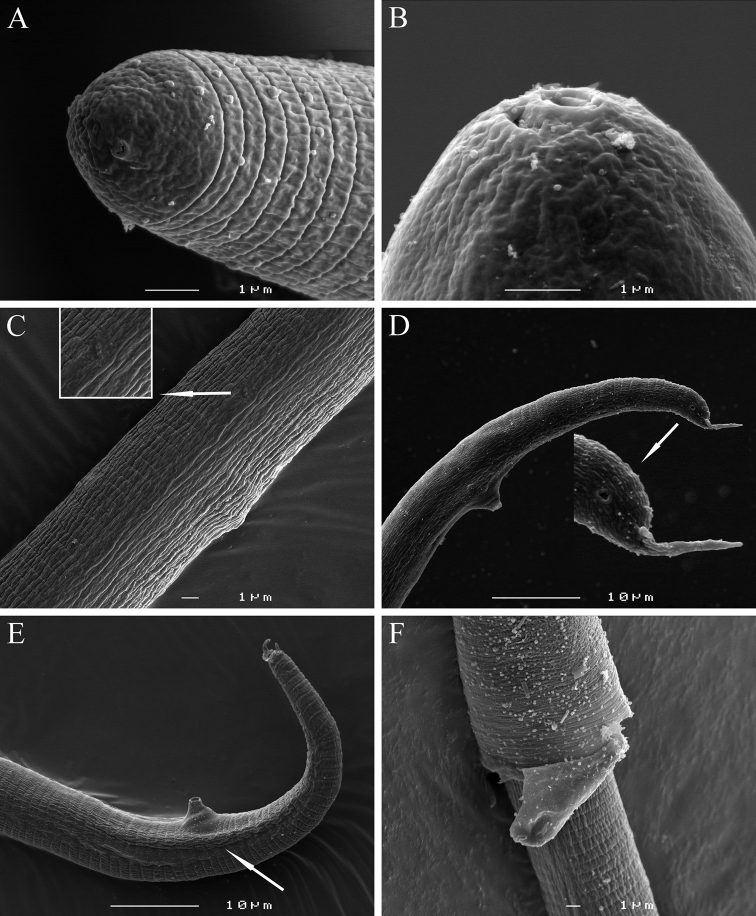
*Meloidoderita salina* sp. n. SEM photographs ofmale**. A, B** Head region **C** Lateral field at S-E pore level (arrow = deirid) **D** Lateral view of tail region(arrow = phasmid) **E** Tail region (arrow = bursa-like structure) **F** Young male within the second-stage juvenile’s old cuticle.

**Figure 8.**
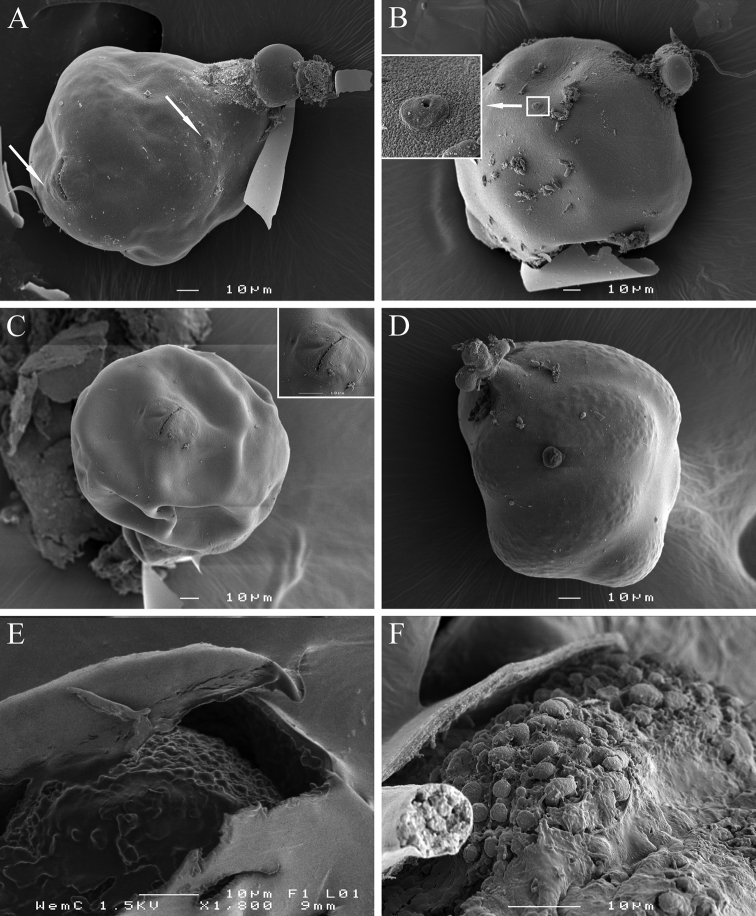
*Meloidoderita salina* sp. n.SEM photographs of female and cystoid. **A** Female body (arrows = S-E pore, anus) **B** Female body (arrow = S-E pore surrounded by cuticular lobes) **C** Vulva and anus **D** Young cystoid with irregular shaped neck region and surface displaying a beaded pattern **E** Sub-cuticular beaded pattern **F** Detail of surface markings in cystoid.

##### Molecular characterization and phylogenetic position of *Meloidoderita salina* sp. n.

**Figure 9.**
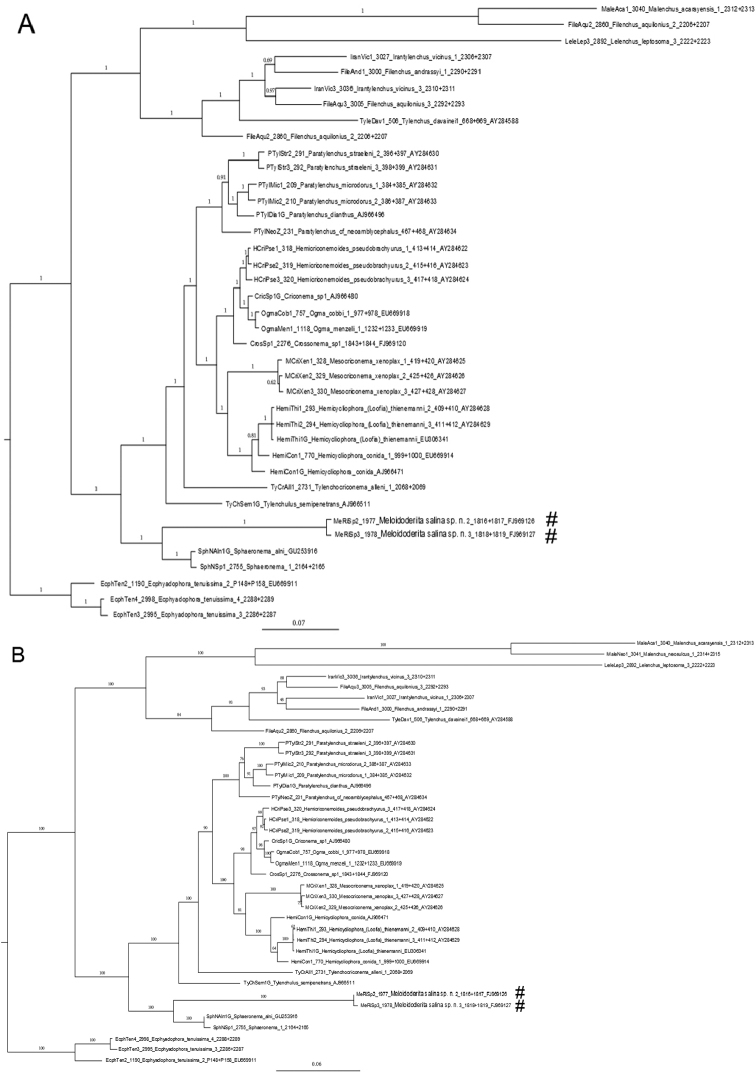
Phylogenetic relationships as inferred from nearly full length of SSU rDNA sequence using GTR + I + G model. Dataset obtained sequences were aligned with the ClustalW algorithm. Numbers near the nodes indicate posterior probabilities in the Bayesian tree (**A**) and ML tree (**B**) as implemented in the program BioEdit 7.0.1. Newly generated SSU rDNA sequences are labeled with a (**#**).

The nearly complete rDNA sequence length of SSU rDNA obtained for *Meloidoderita salina* sp. n. (GenBank FJ969126 and FJ969127) both spanned1728 bp. A local alignment (1883 aligned position) included 39 nearly full length SSU rDNA sequences from related taxa and representatives of the genus *Ecphyadophora* were selected as outgroup. The SSU rDNA sequence analysis and the gene tree represented by the Bayesian and RAxML trees (Fig. 9) revealed a robust sister relationship between the new species and *Sphaeronema alni* within the Criconematina, and the two combined were positioned at the basal part of the local tree. The phylogenetic position of the suborder Criconematina has been analyzed several times (Subbotin 2005, Vovlas et al. 2006, Holterman et al. 2009, van Megen et al. 2009, Palomares-Ruis et al. 2010). However, for conclusive statements on the positioning of this genus among the Criconematina, more rDNA sequence from representatives of the genus *Meloidoderita* are required. Further phylogenetic analyses using SSU rDNA and more taxon sampling are needed to infer intra-generic relationships and the position of *Meloidoderita salina* sp. n. within the Criconematina.

## Discussion

*Meloidoderita salina* sp. n. was described from a salt marsh area at Mont-Saint-Michel Bay in France, parasitizing the halophyte plant *Atriplex portulacoides*. On average, this area has a salinity of about 34–35g/L which usually increases after submersion by the tides. The presence of a well sclerotized S-E duct is a noticeable character, especially in adult males and matured females of *Meloidoderita salina* sp. n. which could be correlated with their saline environment and their halophytic host plant. The presence of a strongly sclerotized S-E duct has been also reported in the genus *Halenchus* N.A. Cobb in M.N. Cobb, 1933 as the only known marine Tylenchomorpha. The genus *Halenchus* with three species is exclusively marine parasitic nematode which produces galls on sea algae (Siddiqi 2000). The “widened and sclerotized excretory duct, exclusively marine, and parasitic on sea algae” are the key characters that have been applied by Siddiqi (2000) in support of the subfamily Halenchinae with its single genus *Halenchus* in Anguinidae Nicoll, 1935 (1926). Considering the sclerotization of S-E duct in both *Meloidoderita salina* sp. n. and *Halenchus*, more physiological studies will probably clarify the role of this structure in these genera.

Spiegel and Cohn (1985) and Vovlas et al. (2006) reported secretion of gelatinous matrix from the vulva slit in *Meloidoderita kirjanovae*. Vovlas et al. (2006) considered it as a discriminating character for differentiation between “*Meloidoderita kirjanovae* and that of other tylenchulids such as *Tylenchulus* and *Trophonema* which secret the gelatinous matrix from the secretory-excretory pore”. They discussed that “this physiological characteristic may confirm the result of phylogenetic analysis” as inferred by Subbotin et al. (2005, 2006) and Sturhan and Geraert (2005), who studied the phylogeny of Tylenchuloidea. Nevertheless, no evidence (e.g. the present of the vulval glands) was observed to support their opinion regarding formation of the gelatinous matrix. In *Meloidoderita salina* sp. n. the S-E pore is a well-developed structure connected to a markedly sclerotized duct running posteriorly. It is possible that this prominent structure could be also involved in the production of the gelatinous matrix.

Poghossian (1966) classified *Meloidoderita* under the family Heteroderidae. However, some years later Kirjanova and Poghossian (1973) established the new family Meloidoderitidae to accommodate *Meloidoderita*, and placed it within the superfamily Criconematoidea. Siddiqi (1985, 2000) proposed the new subfamily Meloidoderitinae to accommodate its single genus, namely *Meloidoderita* and the type species *Meloidoderita kirjanovae*, under the family Sphaeronematidae and the suborder Criconematina on the basis of “the lack of the neck; uterine walls form a protective cystoid body for eggs” (Siddiqi 2000).

Siddiqi (2000) described the genus *Meloidoderita* as mature females with a swollen body, without neck or tail, and males without bursa. Andrassy (2007) also described the *Meloidoderita* adult female as “without neck”. Regardless, Kirjanova and Poghossian (1973), Van den Berg and Spaull (1982), and Golden and Handoo (1984) who reported the presence of an irregularly shaped neck region modified by root tissue and influenced by the cellular root structures. We also observed in *Meloidoderita salina* sp. n. females a well-defined and twisted neck region (Figs 5, 8).

Siddiqi (2000) described the family Sphaeronematidae as “ectoparasite” in which the juveniles “attack and feed on roots ectoparasitically”. However, it was Siddiqi who wrote in 1985: “*Meloidoderita kirjanovae* is reported to be endoparasitic in *Mentha longifolia* roots, becoming secondarily exposed as the growing female ruptures the root epidermis”. Andrassy (2007) also defined the genus *Meloidoderita* as “ectoparasitic” nematodes. In addition to Cohn and Mordechai (1982) and Andrews et al. (1981) who reported *Meloidoderita kirjanovae* and *Meloidoderita* sp. respectively as semi-endoparasitic, Vovlas et al. (2006) recently reported, “Severe infections of *Meloidoderita kirjanovae* were detected on young roots of *Mentha aquatica*. Adult females of *Meloidoderita kirjanovae* protruded from the surface of all infected root segments occurring individually or in clusters, but did not cause distortion of the entire root diameter. Eggs were laid in a gelatinous matrix regularly protruding from the root surface but cystoid body was often located within the root cortex”. Andrews et al. (1981) reported that juveniles migrated intracellularly through the cortex. Further studies are needed to examine the biology, life-cycle and histopathology of *Meloidoderita* sp. and to clarify their parasitic behavior.

Cohen and Mordechai (1982), while studying the biology of *Meloidoderita kirjanovae*, observed several males attached to or enveloped by old second-stage juveniles cuticle. They reported that it “could obviously be identified as offspring of the particular female beneath the egg-mass, rather than having migrated from outside. Furthermore, often more than one molting cuticle was present at the same time, indicating that development of juveniles into adult males was a relatively short process and apparently did not necessitate feeding on the host tissues”. These enveloped males in second-stage juveniles cuticle have been reported by Van den Berg and Spaull (1982). In the present study these enveloped males were also described and we did not observed any J3 or J4 male stages.

In the classification scheme proposed by Siddiqi (2000) the suborder Criconematina was described as “phasmids absent”. Andrassy (2007) has also emphasized that “the absence of phasmids” is one of “the main distinguishing characteristics of this suborder”.

Recently Sturhan and Geraert (2005) assessed the presence of phasmids in Tylenchulidae. They observed phasmid-like structures in *Sphaeronema*, *Meloidoderita*, *Tylenchulus*, *Trophotylenchulus*. However, they did not found phasmids in examined species of Criconematidae, *Hemicycliophora* sp., *Paratylenchus*, *Cacopaurus* and *Tylenchocriconema*. Our observation (LM and SEM) confirmed the presence of phasmids in both juveniles and males of *Meloidoderita salina* sp. n.

Phylogenetic studies done by Subbotin (2005, 2006) Vovlas et al. (2006) Palomares-Ruis et al. (2010) and our phylogenetic analysis showed that *Meloidoderita* together with *Sphaeronema* form a clade and are placed as stem taxa at the base of the Criconematina phylogenetic trees. These morphological observations and molecular studies show that the lack of phasmids in other taxa of Criconematina could be considered as an apomorphic character (Sturhan and Geraert 2005). Hence, within Criconematina those taxa without phasmids could be probably defined by the autapomorphism of the absence of phasmids.

Based on the distribution of the type host *Atriplex portulacoides* in tidal salt marshes in France, it may be expected that *Meloidoderita salina* sp. n. is more widely distributed in West-European salt marshes. Sturhan and Geraert (2005) reported an unknown *Meloidoderita* sp. and also an undescribed *Sphaeronema* species isolated from *Atriplex portulacoides*, both from northern Germany. We suggest further sampling along the North Sea coast (France, Belgium, Germany and UK) to characterize the distribution of this species.

Human consumption is currently one of the most important aspects for cultivation of *Atriplex* spp. It has a salty taste when it is eaten raw or cooked, and is presently served in luxury restaurants. *Atriplex portulacoides* has an important role in primary production, and in the food web in salt marsh ecosystems (Bouchard et al. 1998, Neves et al. 2007, 2008). *Atriplex* spp.is also used for other agricultural and environmental aspects such as dune stabilization, land reclamation, or as livestock fodder and ornamental plant (Aronson 1986, Khan et al. 2000, Daoud et al. 2001). The effect of *Meloidoderita salina* sp. n. on the host plant *Atriplex portulacoides* is unknown and needs to be studied.

It is interesting to report that during this study we found a unique sub-cuticular hexagonal beaded pattern in the cystoids of *Meloidoderita salina* sp. n. This specific pattern can be seen on the surface of the cystoid and displays symmetrical hexagons (Figs 5H, I, 8D–F). This pattern reported in this study is probably the first to be observed among all the identified species of nematodes so far.

## Supplementary Material

XML Treatment for Meloidoderita salina
